# Editorial: Environmental attitudes in context: conceptualisations, measurements and related factors of environmental attitudes

**DOI:** 10.3389/fpsyg.2023.1219471

**Published:** 2023-07-20

**Authors:** Inga Wittenberg, Ghozlane Fleury-Bahi, Oscar Navarro

**Affiliations:** ^1^Center of Sustainability Transformation in Areas of Intensive Agriculture, University of Vechta, Vechta, Lower Saxony, Germany; ^2^Department of Psychology, Nantes Université,, Nantes, Pays de la Loire, France; ^3^University of Nîmes, Nîmes, France

**Keywords:** environmental attitudes, environmental behavior, measurements, context, factors

Climate change remains an important issue nowadays, and the anthropocentric dimension of climate change, that is, the role of human activities, is widely recognized. In this context, pro-environmental behavior and environmental attitudes have been investigated in a large number of studies (e.g., Fujii, 2006; Berenguer, 2007; Patri et al., 2015). Many of these studies aim to understand how to encourage the adoption of environmentally friendly behavior and to analyze the explanatory power of the attitudes developed toward the environment on these behaviors.

Among these studies, different measures and conceptualizations of pro-environmental behavior and environmental attitudes can be found. This implies that numerous attitude scales have been developed to measure such environmental attitudes, which differ in several respects. For example, some measure attitudes toward particular environmental objects such as water consumption (Lam, 1999; Russell and Knoeri, 2019), use of public transport (Heath and Gifford, 2006), or waste separation (Tucker and Speirs, 2010). Other tools aim to measure broader psychological constructs by using an aggregated psychological construct to capture the attitude developed toward a set of environmental behaviors or the general attitude toward the environment.

Social psychology has long been concerned with the link between behavior and attitude. Research results have not shown a clear link between these two dimensions but rather an attitude–behavior gap. The same issue arises when we target pro-environmental behavior more precisely (Claudy et al., 2013; Wyss et al., 2022). It is therefore important to examine this question by considering the different conceptual and methodological approaches in order to identify the reasons for this gap and to figure out which factors need to be considered in order to adopt a more comprehensive approach.

This Research Topic looks specifically at this issue by focusing on different approaches, highlighting the conceptual and methodological diversity related to environmental attitudes.

In line with the aim of this Research Topic, the contributions reflect on different perspectives—theoretical and methodological—and bring together different approaches in the study of environmental attitudes and/or their interplay with different factors in the context of pro-environmental behaviors.

Concerning the theoretical approaches, Urban and Kaiser studied environmental attitudes from the Campbell paradigm perspective, Bazrafkan et al. applied the protection motivation theory, and Zaikauskaite et al. used Hunt–Vitell's moral philosophy-based framework of ethical decision-making to investigate the environmental attitude–behavior gap. Fointiat and Pelt chose the psychosocial engineering model and studied a large number of possible determinants, including the variables of the theory of planned behavior, place identity, sense of community, and a temporal dimension. Jiang et al. studied environmental attitudes and their three components (affective, cognitive, and behavioral), and Bogner and Suarez's study combined environmental attitudes with values and connectedness to nature. The importance of social factors for environmental attitudes and behaviors was studied and highlighted in several articles (Fointiat and Pelt; Jiang et al.; Sun et al.).

There is also a diversity in the methodological approaches chosen to investigate environmental attitudes. More precisely, in three articles (Bogner and Suarez; Sierra Barón and Meneses Baez; Urban and Kaiser), the Rasch model has been applied.

Moreover, some contributions to this topic deal with environmental attitudes in general (Bogner and Suarez; Urban and Kaiser, Zaikauskaite et al.), while others consider them in specific behavioral domains, such as the workplace (Sierra Barón and Meneses Baez), nature preservation (Bogner and Suarez; Bazrafkan et al.), animals' welfare (Jiang et al.), or recycling (Fointiat and Pelt). Interestingly, three contributions deal with pro-environmental behaviors at the farm level, thus considering these behaviors and related attitudes in the context of agriculture. While Bazrafkan et al. applied the protection motivation theory to the use of conservation agriculture by farmers, Sun et al. studied farmers' willingness to participate in this type of agriculture and also their domestic waste classification behavior, thus showing the importance of social factors such as exemplary behavior of relatives and neighbors. Furthermore, in the context of agriculture but from the perspective of public attitudes, Jiang et al. investigated attitudes toward farm animal welfare by assessing the affective, cognitive, and behavioral components. These studies highlight the rising importance of sustainable food production and consumption as part of environmental attitudes and behaviors for both farmers and general society.

These contributions deal with very different cultural contexts, covering China (Sun et al.; Jiang et al.), Iran (Bazrafkan et al.), Cuba (Bogner and Suarez), Colombia (Sierra Barón and Meneses Baez), the United States (Zaikauskaite et al.), Martinique in the French Antilles (Fointiat and Pelt), and a comparison between the 28 EU countries (Urban and Kaiser).

Urban and Kaiser found evidence that the Campbell paradigm represents a sound psychological measurement theory for cross-cultural comparisons. Applying the same scale for the measurement of environmental attitudes among adolescents in Cuba, a low-food-print country, Bogner and Suarez's results also showed good validity of the scale in this context. Sierra Barón and Meneses Baez included work as a context of pro-environmental behavior.

Taken together, the contributions to this Research Topic give insights into attitude–behavior research from different perspectives, illustrating the diversity of factors to take into account and the importance of contextual aspects.

## Author contributions

All authors listed have made a substantial, direct, and intellectual contribution to the work and approved it for publication.
